# Checkpoint and PARP inhibitors, for whom and when

**DOI:** 10.18632/oncotarget.20852

**Published:** 2017-09-12

**Authors:** Jung-Min Lee, James L. Gulley

**Affiliations:** Women’s Malignancies Branch, Center for Cancer Research, National Cancer Institute, Bethesda, Maryland, USA

**Keywords:** immune checkpoint inhibitor, PARP inhibitior, ovarian cancer, BRCA mutation, biomarker

There are now several immunotherapy trials for ovarian cancer although the clinical benefits have been limited to a subset of patients [1]. We recently described preliminary activity and safety of the PD-L1 inhibitor durvalumab, and a PARP inhibitor olaparib, and/or a VEGFR TKI, cediranib in women’s cancer [1]. These combinations provide the opportunity to leverage clinical synthetic lethality where the combined effects of the agents are far more active than either single agent, often leveraging the tumor microenvironment [1, 2].

PARP inhibitors (PARPi) are intriguing combination therapy partners with other pathway inhibitors such as immune checkpoint blockade [2]. The compelling questions in adding immune checkpoint blockade to PARPi in ovarian cancer remain for whom are these agents most active and when should they be used. Benefits of PARPi monotherapy appear greater in women with germline *BRCA* mutation (*gBRCAm*) associated platinum-sensitive recurrent disease [2]. A phase 2 single arm study of durvalumab+olaparib, is now ongoing in *gBRCAm-*associated platinum-sensitive recurrent ovarian cancer (NCT02734004) to examine how clinical benefits by immune checkpoint inhibitors are added to PARPi’s activity in the background of *gBRCAm*. Interestingly, in our study, none of the women receiving durvalumab+olaparib >= 9 months (*n* = 5, median 10.5 months [9-15+]) had germline or somatic mutations in *BRCA* or other DNA repair genes [1].

PARPi blocks DNA repair, resulting in DNA breaks [2]. Fragments of these DNA breaks can enter the cytoplasm and bind to cyclic GMP-AMP synthase (cGAS) leading to an upregulation of the cGAS-STING pathway within the tumor microenvironment, a potent activator of a type I interferons and other immunomodulatory molecules [3]. This may explain why olaparib and talazoparib up-regulate PD-L1 expression in preclinical models [4]. This could ignite or potentiate an anti-tumor immune response. Furthermore, attempts to repair DNA breaks in the tumor by cells with damaged repair pathways could lead to neoantigen formation and subsequent immune recognition. The present study is currently being expanded to a phase 2 study for recurrent ovarian cancer patients with and without *gBRCAm*, in which the role of neoantigen expression and changes in immune microenvironment induced by PARPi will be further examined. The optimal selection of patients for treatment with PARPi and immune checkpoint inhibitor in non-*BRCA* mutated settings and better understanding of the mechanisms of action will require further characterization and analysis.

Immunotherapy manifests differently from traditional chemotherapy, eliciting delayed response kinetics [5]. It has been proposed that immunotherapy may be more effective in patients with lower tumor burden, in whom disease progression may be less rapid, thereby allowing ample time for the immunotherapy to evolve [5]. In addition, immunotherapy may be more efficacious in patients when administered earlier during the disease course, correlative with a more intact immune system capable of responding to an exogenous immunotherapy [6]. Our preliminary data also suggest durvalumab +olaparib may be more effective in ovarian cancer with lower tumor burden and no ascites [1]. It has been known that regulatory T (Treg) cells suppress autoreactive T cells, preferentially accumulates in ascites, and correlate with poor clinical outcome in ovarian cancer [7]. Future use and clinical trials should take into consideration that immunotherapies may elicit a better immune system response if used while the patient is still immunocompetent with earlier stage of disease course, and lower tumor burden.

The overexpression of PD-L1 is an important and widely explored biomarker for response to immune checkpoint inhibitors. However, PD-L1 expression by immunohistochemistry fails to accurately select all patients suitable for PD-1/PD-L1 inhibitors [1]. Recently, a new classification of tumors has been proposed based on PD-L1 status and the presence or absence of TILs; type 1, PD-L1+/TILs+ called ‘immune resistant’ driving adaptive immune resistance; type 2, PD-L1-/ TIL- indicating ‘immune ignorance’; type 3, PD-L1+/ TIL- indicating ‘intrinsic induction’ related to oncogenic induction of PD-L1 rather than TILs driven; and type 4, PD-L1- /TIL+ called ‘tolerant tumors’ indicating the role of other suppressor(s) in promoting immune tolerance [8]. The presence of both TILs and PD-L1 in the tumor microenvironment could indicate an adaptive immune resistance to endogenous antitumor activity, suggesting that tumors with PD-L1+/ TILs+ would probably be more sensitive to treatment with PD-1/PD-L1 inhibitors [8]. This tumor microenvironment type suggests that TILs play a more crucial role in predicting response to PD-1/ PD-L1 inhibitors than constitutive PD-L1 positivity. This classification could be useful in stratifying patients to be treated with immune checkpoint inhibitor combinations.

The advent of immunotherapy combination therapy presents us with new approaches in ovarian cancer treatment with promising outcomes, preliminarily. Multiple clinical trials are currently being conducted to better define the role of PARPi and immunotherapy combinations, and further investigation is warranted to develop and identify predictive biomarkers. Assessing how immunotherapies should be incorporated with current standard-of-care treatments, such as PARPi is essential to make progress in the treatment of ovarian cancer.
